# Human Adenovirus Gene Expression and Replication Is Regulated through Dynamic Changes in Nucleoprotein Structure throughout Infection

**DOI:** 10.3390/v15010161

**Published:** 2023-01-05

**Authors:** Morgan R. Jennings, Robin J. Parks

**Affiliations:** 1 Regenerative Medicine Program, Ottawa Hospital Research Institute, Ottawa, ON K1H 8L6, Canada; 2 Department of Biochemistry, Microbiology and Immunology, University of Ottawa, Ottawa, ON K1N 6N5, Canada; 3 Centre for Neuromuscular Disease, University of Ottawa, Ottawa, ON K1N 6N5, Canada; 4 Department of Medicine, The Ottawa Hospital, Ottawa, ON K1H 8L6, Canada

**Keywords:** adenovirus, epigenetics, nucleoprotein, nucleoprotein remodeling, viral replication, epigenetic modulators

## Abstract

Human adenovirus (HAdV) is extremely common and can rapidly spread in confined populations such as daycare centers, hospitals, and retirement homes. Although HAdV usually causes only minor illness in otherwise healthy patients, HAdV can cause significant morbidity and mortality in certain populations, such as the very young, very old, or immunocompromised individuals. During infection, the viral DNA undergoes dramatic changes in nucleoprotein structure that promote the rapid expression of viral genes, replication of the DNA, and generation of thousands of new infectious virions—each process requiring a distinct complement of virus and host-encoded proteins. In this review, we summarize our current understanding of the nucleoprotein structure of HAdV DNA during the various phases of infection, the cellular proteins implicated in mediating these changes, and the role of epigenetics in HAdV gene expression and replication.

## 1. Introduction

Adenoviruses (AdV) are a diverse group of viruses that infect a large range of vertebrates, including humans [1]. Human AdV (HAdV) was first isolated from adenoid tissues (from which it derives its name) in the 1950s [2,3]. Since its discovery, research into HAdV has contributed greatly to our general understanding of many aspects of molecular cell biology, largely due to the fact that viruses are exquisitely adept at identifying crucial pathways/processes within the cell and then modulating the activity of key proteins within those pathways to facilitate optimal viral replication. For HAdV, examples include gene splicing, which was first identified as an aspect of HAdV gene expression [4,5], but subsequently shown to be a fundamental process for almost all genes in eukaryotic cells. Studies of cellular proteins that interact with E1A proteins (early region 1A, the first viral proteins expressed following HAdV infection) contributed to the identification and/or functional characterization of many key cellular proteins, such as the histone acetyltransferase E1A-associated protein p300 (EP300 [6,7]), and the tumor suppressor Rb and its family members [8,9,10]. In many ways, studying virus biology is synonymous with studying host molecular cell biology.

Within the cell, eukaryotic genomic DNA is packaged into chromatin, a highly organized complex of DNA and proteins that encodes epigenetic information governing gene expression and cell identity [11,12,13]. The basic unit of chromatin is the nucleosome, 147 base pairs of DNA wrapped around two molecules each of H2A, H2B, H3, and H4. Histones play an active role in gene regulation, through post-translational modification (PTM) of their N-termini, which are subsequently recognized by many cellular regulatory proteins [12,14]. As a nuclear virus, it is within this environment that HAdV must express its genes and replicate its DNA, suggesting that the virus has likely evolved an ability to manipulate cellular epigenetic pathways to effectively accomplish these tasks. The purpose of this review is to summarize our current knowledge of viral and host proteins that establish and remodel the HAdV nucleoprotein structure throughout the various stages of infection, to promote optimal viral gene expression and replication.

## 2. Adenovirus Biology

**Figure 1:**
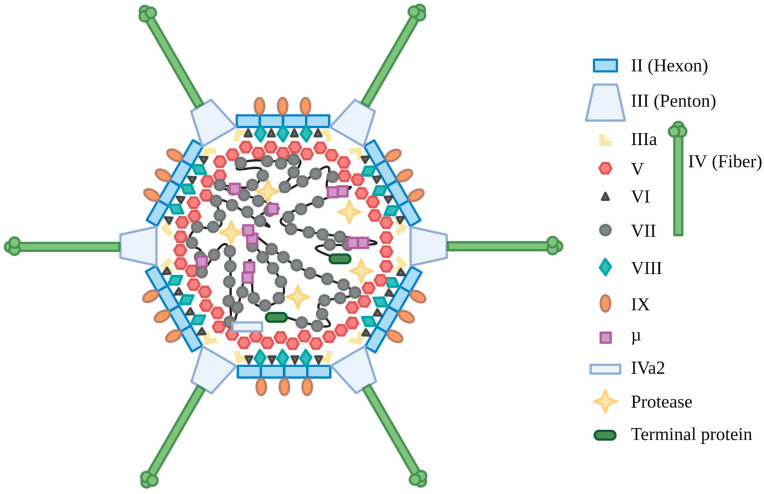
Schematic of the HAdV-5 virion. Adapted from [38], with modifications based on [33,39,40,41]. Created with BioRender.com.

The AdV virion (Figure 1) is comprised of a non-enveloped icosahedral capsid with a diameter of ∼80–90 nm, with each spike-like fiber protein protruding an additional ~12–35 nm, depending on the specific subtype [15]. HAdV contains a linear double-stranded DNA (dsDNA) genome of ∼30–40 kb [15], with over 100 distinct types grouped into species A-G [16]. HAdV types 2 (HAdV-2) and 5 (HAdV-5), which both belong to species C, are perhaps the most extensively characterized and are very similar in biology and DNA sequence (i.e., most findings utilizing HAdV-2 are directly applicable to HAdV-5 and vice versa). If not otherwise stated, we refer here primarily to the situation in HAdV-2 and HAdV-5. The HAdV-5 genome is approximately 36 kb and encodes over 40 proteins. Adenoviral coding regions are designated early or late depending on when they are expressed (i.e., before or after DNA replication) [17]. The E1 region encodes the E1A proteins, which induce mitogenic activity in the host cell and stimulate expression of other viral genes [18,19], and E1B proteins, which serve to prevent apoptosis induced by the activities of E1A [20,21]. E1B-55K also associates with E4-encoded proteins and other cellular proteins to form a ubiquitin ligase complex that promotes degradation of specific proteins, such as the cellular Mre11-Rad50-Nbs1 (MRN) DNA repair complex [22]. The E2 region encodes proteins involved directly in viral DNA replication [23]. The E3 region encodes proteins that primarily have immunomodulatory functions [24], but also encodes the adenovirus death protein (ADP), which assists with cell lysis at the end of the infection cycle [25]. The E4 region encodes proteins with numerous functions, such as alteration of cellular protein stability, reorganization of cellular structures within the nucleus, regulation of late viral RNA splicing, and inhibition of the cellular DNA damage response (DDR), with several of these proteins having redundant activity [26,27]. The onset of viral DNA replication leads to the activation of the major late promoter (MLP) [28], which gives rise to a long transcript that is alternatively spliced into the late transcription units, L1–L5. Indeed, during infection, HAdV uses high levels of alternative splicing, generating over 11,000 differently spliced transcripts, likely as a mechanism driving evolutionary change and possibly compensating for the reduced inherent spontaneous mutation rate during genome replication of this DNA virus relative to many RNA viruses [29,30]. The regions encoding the L4-22K and L4-33K proteins are initially expressed at low levels from a promoter located within the L4 region [31], and these proteins contribute to full activation of the MLP [32]. Additional transcripts are produced at intermediate/late times of infection, including the structural protein IX (pIX) and the IVa2 protein, which is involved in packing the viral DNA into immature virions [33]. The U exon protein (UXP) is expressed late in infection and likely has a role in DNA replication or RNA transcription, but it is largely uncharacterized [32,34]. HAdV also encodes two virus-associated (VA) RNAs (VA RNAI and RNAII) which improve the translation of early and late viral genes, inhibit activation of the interferon response, and may alter the expression of host genes [35,36,37]. Given the large number of transcripts produced during infection [30], there are likely other as-yet uncharacterized proteins generated during normal HAdV infection. Located on both ends of the genome are the inverted terminal repeats (ITRs), which vary in length based on type (e.g., ~100 bp for HAdV-C5), and act as the origin of DNA replication, with the ~200 bp viral DNA packaging sequence positioned adjacent to the left ITR.

The mature HAdV-5 capsid is composed of proteins encoded within the late region. There are three major (II, III, and IV) and five minor (IIIa, IVa2, VI, VIII, and IX) proteins that make up the viral capsid [33,38,39,40,42], reviewed in [15]. Trimers of protein II (more commonly known as hexon) form each facet of the icosahedron, with vertices being capped by pentamers of protein III (also known as penton). Extending from the penton bases are trimers of protein IV, the fiber protein. In large part, the minor polypeptides function to stabilize the major proteins and solidify the virion structure [43,44]. HAdV DNA within the mature capsid associates with three highly basic proteins, VII, V, and μ (Mu) [42,45,46,47,48,49]. The actual architecture of the HAdV DNA core within the virion is currently unknown [15]. Protein VII is the main protein responsible for wrapping and condensing the viral DNA [50]. Digestion of the viral nucleoprotein core with micrococcal nuclease (MNase) produces protected fragments of 90–150 bp [51,52], somewhat analogous to what is observed for cellular chromatin. The protein VII-DNA nucleoprotein complex is believed to be organized into a central dense core with 12 large spherical nucleoprotein projections, termed adenosomes, which extend into each vertex of the capsid [53,54]. Recent work using atomic force microscopy revealed that protein VII condenses the viral genome by both direct clustering and by promoting bridging of different regions through protein Mu [41]. Virions formed in the absence of protein VII are non-infectious due to an inability to escape the endosome as a result of a failure to process pre-protein VI [55], which normally facilitates endosomal escape during infection [56]. Protein V forms a shell around the protein VII-DNA complex within the virion [53,57,58] and tethers it to the capsid [47,57,58,59,60]. Virions formed in the absence of protein V are recoverable but less infectious due to premature capsid degradation, which leads to early release of the viral DNA into the cytoplasm [61,62]. Inappropriate release of the viral DNA in the cytoplasm can activate antiviral innate immune signaling through engagement of the cytoplasmic DNA sensor cGAS [62], clearly illustrating the crucial nature of the virion-stabilizing function of protein V.

The HAdV genomic DNA may also contribute indirectly to virion stability, as virions with genomes less than ~80% of the wildtype genome length are relatively unstable and rapidly disintegrate upon heating [63,64]. Indeed, HAdV strongly selects for appropriate genome size: vectors with genome sizes greater than 105% [65] or less than 75% [66] undergo spontaneous genome rearrangement to attain a size closer to wildtype virus. This phenomenon has been observed for other viruses with icosahedral capsids [49,64]. For HAdV, the mechanism by which the genome size influences virion stability has not been elucidated. In the HAdV virion, the nucleoprotein core extends into the vertex regions of the inner capsid and makes direct contact with penton and peripentonal hexon [59]. This region appears to be preferentially destabilized upon heating of HAdV vectors with sub-wildtype genomes [63]. Tight packaging of wildtype-length DNA into the capsid may force the DNA in the vertex regions into the proper position to achieve the required linkage between the DNA and the peripentonal hexons, bridged by proteins V and VI. For virions containing sub-wildtype length genomes, loose packing of the nucleoprotein core may prevent these interactions, leading to destabilization of the capsid.

While the receptors used by HAdVs for cell entry vary by type, for HAdV-5, the fiber protein binds to the Coxsackie adenovirus receptor (CAR), the primary receptor for HAdV-5 and Coxsackie B virus [67,68]. HAdV-5 can also utilize heparin sulfate proteoglycans located on the cell surface as an alternative receptor, either through direct binding to the fiber shaft of HAdV [69] or bridged through the interaction of HAdV-5 with various factors in the blood [70,71,72,73]. After the initial binding to CAR, the HAdV-5 penton protein interacts with a secondary receptor composed of αvβ3 or αvβ5 integrins, which triggers internalization of HAdV and occurs through clathrin-mediated endocytosis [74]. Acidification of the endosome alters the HAdV capsid structure, allowing for the release of protein VI from the inner capsid. Protein VI possesses membrane lytic activity [56] and mediates rupture of the endosome and release of the virus [75]. As the capsid is transported to the nucleus along the microtubule network [76], it is progressively dismantled [77,78,79]. Upon reaching the nuclear pore, protein V is released from the viral genome following ubiquitination by the E3 ligase Mind bomb 1 [62,78], which allows release of the protein-VII-wrapped HAdV DNA and subsequent import into the nucleus [62,77,78,79,80,81,82]. Both viral DNA replication and progeny formation occur within the nucleus, and one infectious cycle takes 24–36 h in immortalized cells, although the time for completion of the lifecycle is slightly extended in primary cells.

## 3. The Role of Protein VII in Early Infection

Protein VII is the only viral protein that accompanies the viral DNA into the nucleus and appears to have several functions during early infection. Protein VII is thought to protect the incoming HAdV DNA from detection by the DDR [83]. Inadvertent activation of the DDR can lead to concatemerization of the viral DNA, which presumably is too large to be efficiently packaged into capsids [22,84]. Although protein VII is important for protecting the viral DNA during the very early stages of virus infection, some studies have suggested that prolonged association could negatively impact early viral gene expression. When injected into Xenopus laevis eggs, protein VII condenses the Xenopus chromatin and inhibits transcription [85]. Cell-free systems developed to study HAdV DNA replication have shown that protein-VII-wrapped viral DNA allows for only limited transcription and DNA replication [86,87,88]. Conversely, other studies have suggested that protein VII may be important for actually promoting transcription of viral genes. Transfection of plasmid DNA complexed to protein VII actually enhanced reporter gene expression compared to naked DNA alone [89]. The N-terminus of E1A is capable of associating with protein VII, which might allow protein VII to recruit E1A and other E1A-associated proteins, such as components of the cellular transcriptional machinery, to the protein-VII-wrapped DNA to facilitate initiation of transcription [85,90]. Thus, dynamic regulation of protein VII is likely necessary for optimal viral growth; sufficient protein VII must be removed or remodeled to decondense the viral DNA–nucleoprotein complex to allow access to the transcription machinery, but some protein VII may need to be retained to stimulate transcription.

Protein VII can undergo PTM, which influences its subcellular localization [91,92]. For example, mature protein VII can be acetylated on lysine 2 or lysine 3, and the mutation of either of these residues to alanine changes the localization of protein VII from being dispersed throughout the chromatin to localized in the nucleolus [92], while the same change on pre-protein VII has the opposite effect [91]. In the case of cellular histones, acetylation of histones “loosens” the cellular chromatin structure and promotes transcription [14], but whether such modification is required to loosen the viral nucleoprotein structure or as a prelude to protein VII removal is unknown.

The conflicting data on the importance of protein VII for early transcription are mirrored by conflicting data on the timing of protein VII removal from the viral DNA. Protein VII can be cross-linked to the viral DNA at all stages of infection [47], and chromatin immunoprecipitation (ChIP) studies have shown that protein VII can be found bound to the viral DNA up to at least ~10 hpi [85,89,93,94,95,96]. However, some studies found that protein VII association does not change during the early stage of infection [47,90,94], while others instead suggest a decline in protein VII association with the viral DNA [89,93,95,97]. Compounding this issue is the observation that protein VII association may vary depending on the region of the genome being analysed. For example, between 1 and 10 hpi, protein VII appeared to remain stably associated with the late-gene hexon coding region but showed declining association over time with the major late promoter [89]. Protein VII also showed reduced association with the late hexon region when comparing early (4 hpi) versus late (18 hpi) time points of infection [96]. 

Another aspect of protein VII biology with contradictory data is whether transcription of the HAdV DNA template is required for removal of protein VII. Some studies showed that inhibiting transcription prolongs the retention of protein VII on the HAdV genome [90,97], while others saw no such effect [89,95,96]. Recruitment of E1A to the protein-VII-wrapped genome may stimulate early transcription, which could subsequently strip protein VII from the viral DNA [85,90]. However, protein VII is still removed in the absence of E1A [89,95], suggesting that this function of E1A is not essential for expression of virus-encoded genes. If E1A was absolutely required for transcription initiation on HAdV DNA, HAdV-based vectors that are deleted of the E1 region would not function well, which is not the case. Transgene expression from E1-deleted vectors appears quite efficient and can be detected as early as 2 hpi [95]. However, there may be host-encoded proteins that can compensate for the loss of E1A. Indeed, E1-deleted HAdV can complete a full replication cycle in certain cell types, although the time required to complete the replication cycle is extended [98,99], suggesting that compensating proteins may exist. 

Based on studies in cell-free systems, three cellular proteins have been implicated in remodeling the protein-VII-wrapped viral DNA. Transcription activating factor (TAF)-Iβ (also known as SET) is perhaps the best characterized [87]. TAF-Iβ can form a tertiary complex with the protein-VII-wrapped DNA [93,94,100], which improved accessibility to restriction enzymes [88], which would presumably also increase accessibility to transcriptional activators. Fluorescently labeled TAF-Iβ co-localizes with protein VII on incoming viral genomes in the nucleus, which can be used to track the virus in live cells [101]. siRNA-mediated knockdown of TAF-Iβ had a modest effect on viral replication [102] but did not affect the binding level of protein VII on viral DNA as assessed by ChIP [89], suggesting that other proteins are responsible for the removal of protein VII or can compensate for the lack of TAF-Iβ. TAF-II (NAP-1 [103]) and TAF-III (B23/nucleophosmin [104]) were also identified as potential remodelers of the protein-VII-wrapped viral DNA in cell free systems, and both can stimulate replication from the HAdV core, while TAF-II can also enhance transcription.

Protein VII can also associate with host chromatin, where it contributes to suppressing immune danger signals [92]. Protein VII was shown to interact with the high-mobility-group B (HMGB) family of proteins and cause their retention on chromatin [92]. Since free HMGB functions as a danger signal to activate an immune response [105], these data suggest that protein VII is acting to blunt the cells’ response to infection. Protein VII also causes a depletion of histone H1 isoforms from cellular chromatin [106]. This imbalance of HMGB and H1 caused by protein VII prevents cell cycle progression, which the authors speculated could be a mechanism normally employed by HAdV to ensure that the cell cycle progression induced by E1 proteins stops in S phase [106], as progression beyond this phase may be deleterious to viral replication. Indeed, in the absence of protein VII, HAdV-infected cells express markers of G2 and mitosis [106]. Thus, protein VII released from the incoming virion, or newly synthesized within the infected cell, may help shape the microenvironment for optimal viral replication.

## 4. HAdV DNA Associates with Nucleosome-like Structures Early in Infection

Though there was initially debate over whether cellular histones were found on HAdV DNA [107,108,109,110,111] https://www.ncbi.nlm.nih.gov/pmc/articles/PMC3315334/ (accessed on 9 December 2022)—gkr1076-B59, more recent studies have clearly shown that histones are detectable on the HAdV DNA as early as 1 h post-infection [89,95,96,112]. Within the host cell, histone variant H3.1 is expressed exclusively during S phase and is deposited on newly replicated cellular DNA by the Chromatin Assembly Factor I (CAF1) complex [113,114]. Thus, deposition of H3.1 is intimately tied to host DNA replication. In contrast, the histone variant H3.3 is expressed throughout the cell cycle and is deposited on DNA independent of DNA replication [115]. The histone chaperone involved in deposition of H3.3 varies by chromosome region; H3.3 is deposited on active promoters and enhancers by EP400 [116], within actively transcribed genes or on incoming male pro-nuclear DNA by the histone cell cycle regulator A (HIRA) complex [117,118], and on telomeres and pericentric DNA repeats by death-associated protein 6 (DAXX) [117,119,120]. Thus, the identity of the H3 variant contained in nucleosomes can provide some insight into the mechanism of deposition. During normal viral transmission, HAdV primarily infects non-dividing cells, inducing cell cycle progression only after E1A gene expression has already occurred, suggesting that HAdV DNA is likely to be chromatinized by exploiting a mechanism independent of host cell or viral DNA replication. ChIP experiments showed that DNA from replication-defective HAdV-based vectors [95] and wildtype HAdV [89,96,112] preferentially associates with H3.3 beginning as early as 1 hpi. Although the H3.1 chaperone CAF-1 appears to localize to viral replication centers in the nucleus of infected cells [112], H3.1 does not associate with HAdV DNA at any point during infection [95,96,112]. Herpes Simplex Virus 1 (HSV1) DNA also associates preferentially with H3.3 during the early phase of infection, although HSV1 switches to association with H3.1 late in infection [121]. Knockdown of HIRA decreased association of H3 with the HAdV-based vectors [95] and HSV1 DNA [121] and subsequently reduced expression of genes encoded by the viruses. However, knockdown of HIRA did not decrease association of H3.3 on wildtype HAdV-5 DNA [96], suggesting that either HIRA is not responsible for depositing H3.3 on wildtype HAdV DNA or that there are other proteins that can compensate for the loss of HIRA for wildtype virus but not for HAdV-based vectors. DAXX is likely not used by HAdV as a histone chaperone, as it is actively degraded during infection [122]. HAdVs that are unable to degrade DAXX ultimately associate with unacetylated histones, resulting in repression of viral transcription [122,123,124], indicating that DAXX functions as an innate factor to limit gene expression on incoming viral DNA. Despite TAF-Iβ demonstrating histone chaperone activity [88], it does not appear to act as a histone chaperone for incoming HAdV genomes [89]. Knockdown of the histone chaperone CHD1, which binds to trimethylated lysine 4 on H3 (H3K4me3), a mark associated with active genes [125], did not lower viral yield in HAdV-5-infected cells [126], suggesting it is not used by HAdV to remodel its DNA.

Work by Komatsu et al. [89] demonstrated that HAdV DNA associates with all members of the nucleosome, H2A–H2B and H3–H4. While H4 would be deposited as a heterodimer with H3.3, the chaperone responsible for deposition of H2A/H2B is unknown. Upon MNase digestion, HAdV DNA in the nucleus displays a repeating ∼200 bp structure similar to cellular chromatin, suggesting that much of the DNA is indeed wrapped in physiologically spaced, nucleosome-like structures, at least during the early phase of infection [95,107,108,109,110,127]. All regions of the genome are represented in the MNase-protected fractions [107]. The observation that both protein VII and histones can be found bound to the same viral DNA molecule at the same time suggests that the viral chromatin likely does not completely resemble that of the host cell [89]. At late times of infection (16–18 hpi), viral genomes no longer appear to be wrapped in the repeating nucleosome-like structure [96], instead showing irregularly spaced nucleosome-like structures at approximately one-tenth the density of cellular chromatin (three versus twenty-six nucleosomes per µm of DNA, respectively [109]). HSV1 also shows viral DNA associating with regularly spaced nucleosomes during latent infection, while the spacing becomes ‘unstable’ during a lytic infection [128]. For HAdV, the decreased nucleosome density at late times of infection could be merely a result of decreased histone expression [129,130] coupled with increased amounts of viral DNA, thereby reducing the pool of histones available for binding to the viral DNA.

## 5. Nucleoprotein Structure of HAdV during Active DNA Replication

HAdV has evolved a unique mechanism to maintain genome length and replicate the ends of its linear dsDNA genome. Proteins directly involved in viral DNA replication are encoded within the E2 transcription unit, which consists of two regions, E2A and E2B, which have separate polyadenylation sites [23]. E2A encodes the 72 kDa DNA-binding protein (DBP), whereas the E2B region codes for the 80 kDa precursor terminal protein (pTP) and the 140 kDa HAdV DNA polymerase (Pol). Replication initiates through a protein priming mechanism (reviewed in [131]); Pol covalently attaches a dCMP residue to pTP, the pTP/dCMP molecule then binds to the viral ITR, and the 3’-OH group of the dCMP serves as a primer for elongation by Pol. Thus, the dCMP moiety becomes the first nucleotide of the newly synthesized genome. Pol replicates one entire strand of the genome in a continuous manner, while the second strand is displaced. pTP remains covalently bound to the ends of the viral DNA at all stages of infection and in the virion but is cleaved by the HAdV-encoded protease to produce the mature 55 kDa terminal protein (TP) during virion maturation. DBP aids in unwinding the dsDNA genome during replication [132,133] and promotes DNA replication elongation [134]. DBP also coats and protects the displaced single-stranded DNA (ssDNA), thus preventing immune and DNA damage responses that can be induced in the cell against naked DNA [135,136]. The displaced ssDNA can then form a “pan-handle” structure (by annealing the complementary ssDNA ITR at each end of the molecule), which functionally resembles the native ITR and can serve as a template for initiation of replication of the ssDNA in a manner similar to the dsDNA genome [137]. DBP also binds dsDNA but with lower affinity [134]. It is unlikely that newly replicated HAdV DNA is “naked” within the nucleus at any point in the lifecycle; naked DNA is not well tolerated in the nucleus of a cell [138] and can contribute to genome instability and susceptibility to mutation. Since there is a dramatic decline in cellular histone synthesis late in HAdV infection [129,130], DBP may be the major protein associating with viral replicative intermediates and newly replicated genomes within the cell at late times of infection. However, DBP-wrapped viral DNA is probably not the template for late gene expression [139,140], as cellular RNA polymerase likely cannot contend with such a radically different nucleoprotein structure compared to cellular chromatin. A small fraction of the viral DNA does remain associated with H3.3 at 18 hpi [96], and it is perhaps this subpopulation that acts as the transcription template. Indeed, each genome in a replication center may associate with different proteins depending on its assigned task—histones for gene expression, DBP for genome replication, and pVII for packaging into capsids, as discussed below.

## 6. The Switch from Histones to Protein VII

Prior to encapsidation, the histones or DBP must be displaced from the HAdV DNA and replaced with pre-protein VII. However, the mechanism by which this occurs is unknown. Given that histone production is reduced late in HAdV infection [129,130], as the levels of cellular histones decline and the quantity of pre-protein VII within the cell increases, the viral genome may be predisposed to associate with pre-protein VII rather than histones [108,110,141]. During late infection, newly replicated viral dsDNA accumulates within the center of “virally induced post-replicative” (ViPR) bodies along with pre-protein VII, and these regions actively exclude cellular histones, indicating another potential mechanism for this switch [142]. Additionally, as discussed above, PTM of both pre-protein VII and protein VII may influence their localization within the nucleus [91,92] and may therefore be another mechanism used by HAdV to mediate the switch of histones to pre-protein VII on viral DNA. 

Mixing HAdV DNA and purified pre-protein VII leads to the formation of an insoluble complex [143], suggesting that a specific cellular chaperone(s) is required for deposition of pre-protein VII on HAdV DNA. TAF-III, identified using a cell-free system as a HAdV-core remodeler, has a greater affinity for pre-protein VII than the mature protein VII and will transfer pre-protein VII onto HAdV DNA in cell-free systems [144]. Consistent with this, depletion of TAF-III caused histones to be retained longer on the viral DNA [145]. Pre-protein VII also interacts with chromatin remodeling proteins CHD3 and BAZ1A, suggesting these proteins could also be involved in late viral chromatin remodeling [146], although this has not been confirmed experimentally. Once wrapped in pre-protein VII, the viral DNA is available for packaging into capsids. Based on current evidence, HAdV capsids appear to form around nascent viral cores, as opposed to viral DNA being inserted into partially assembled capsids (the concerted vs. sequential models, respectively) [147].

## 7. Epigenetic Regulation of Viral and Cellular Chromatin during HAdV Infection

The observation that HAdV DNA is wrapped in physiologically spaced nucleosome-like structures, at least at earlier times of infection, suggests that HAdV may make use of epigenetic regulation to help modulate viral gene expression. HAdV certainly induces global changes in the epigenetic landscape of the host cell in order to modulate the microenvironment for optimal viral replication. E1A interaction with CBP and p300 causes a global reduction in histone H3 lysine 18 acetylation (H3K18ac, a histone mark for active genes) [148] but maintains H3K18ac marks on the promoters for genes involved in the cell cycle [149]. Kulej et al. [150] also observed large-scale global changes in histone PTMs over the course of infection, but these changes were not mapped to any specific regions or genes in their study. Epigenetic changes are also observed on the HAdV chromatin, with an increase in the association of acetylated H3 at all HAdV promoters tested [89]. Since acetylated histones are commonly associated with actively transcribed genes, it suggests that as these promoters become active, they adopt an epigenetic status similar to cellular genes, which may aid in recruiting appropriate co-factors for optimal gene expression. 

Several studies have demonstrated that compounds that modulate the activity of cellular epigenetic regulatory proteins are capable of impacting HAdV replication (reviewed in [151]). Histone deacetylase (HDAC) inhibitors, such as valproic acid [152], suberoylanilide hydroxamic acid (SAHA, also known as vorinostat), and trichostatin A [153], can inhibit HAdV infection. Indeed, HDAC inhibitors appear effective against multiple HAdV types, including HAdV-B7, HAdV-C5, and HAdV-E4 [153]. A screen of small molecules that modulate the activity of epigenetic regulatory proteins identified numerous pan-HDAC inhibitors with anti-HAdV properties [154]. However, HDAC inhibitors were also shown to reactivate latent viruses in tonsillectomy specimens [155], suggesting a varied mechanism of gene regulation during lytic and latent infection. Histone methyltransferases (HMTs) also appear to be required for HAdV replication, as inhibition of EZH2/1 (an H3K27 HMT [156]) or treatment of cells with chaetocin (an inhibitor of H3K9 HMT G9/G9a and SUV39H1/2 [154]) negatively impacted HAdV replication. These H3K9 methylation marks are typically associated with gene repression [157], and it is not clear if these compounds are primarily impacting viral or cellular chromatin (or both). In the case of the EZH2/1 HMT, inhibition appears to be affecting cellular chromatin and promotes the expression of cellular genes that establish an antiviral state within the cell that is hostile to virus replication [156].

## 8. Conclusions

**Figure 2:**
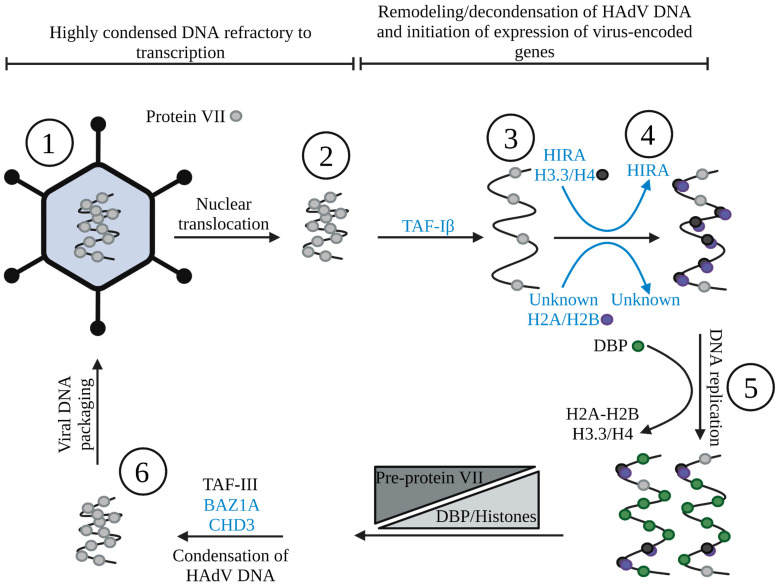
The structure of HAdV DNA throughout the replicative cycle. Black represents events/processes we know, while blue represents speculation based on the literature. Within the viral capsid, condensed viral DNA is wrapped in protein VII [50] (1). After entry into the cell, the protein-VII-wrapped DNA is translocated into the nucleus [77,78,79,80,81,82] (2) and is subsequently remodeled, possibly involving TAF-Iβ [87], removing some protein VII from viral DNA and decondensing the genome (3). Cellular histones are deposited onto viral DNA [89,95,96,112], possibly by HIRA [95] and other histone chaperones (4), allowing for more efficient early gene expression. The nucleosome-like structures present on the viral DNA contain H2A-H2B, H3.3, and H4 [89,96]. Following DNA replication, the viral DBP is deposited onto replicating genomes [134,135], while some histones are present for late gene expression [96], possibly on two distinct DNA populations (5). As late infection progresses, histone pools decrease and are gradually replaced on viral DNA by pre-protein VII by TAF-III [145], recondensing the genome (6). The condensed viral core is subsequently encapsidated [147] and the virion released following lysis of the cell (1). Created with BioRender.com.

As we have shown throughout this review, the HAdV nucleoprotein structure undergoes dynamic changes throughout viral replication (summarized in Figure 2). Understanding the mechanisms by which HAdV accomplishes viral gene expression and replication in the host cell is important for several reasons. First, while typically self-limiting in healthy adults, HAdV can induce severe disease in certain populations, such as pediatric, geriatric, and immunocompromised individuals (including those on immunosuppressants) [158,159,160,161]. For example, recently, HAdV has received significant notoriety for its potential involvement in a mysterious outbreak of severe acute hepatitis of unknown origin that has been diagnosed in over 650 children from 33 countries around the world (reviewed in [162,163]). Despite the potential for significant HAdV-induced disease in infected patients, there are still no approved therapeutics for HAdV [151]. Use of some broad-spectrum antivirals shows some success against HAdV [164], but there are often secondary toxicities associated with these treatments [165]. Epigenetic modulator compounds, such as HDACs, are already being explored for use against other diseases such as cancer; thus, if they are proven safe, they could easily be used to treat HAdV infection [151]. Second, a better understanding of the dynamics of the epigenetic regulation of HAdV may help improve the safety and efficacy of HAdV-based vectors for gene therapy, such as improving tissue-specific transgene expression to avoid effects in non-target cells [166,167]. Finally, given that HAdV is adept at identifying and hijacking key cellular processes, exploration of HAdV biology can lead to greater knowledge of host molecular cell biology, knowledge that could ultimately lead to new treatments and therapies for human disease. Undoubtedly, continued studies of HAdV-mediated epigenetic regulation of viral and cellular gene expression should provide novel insight in all of these areas.
